# Comparison of clinical and biochemical markers of dehydration with the clinical dehydration scale in children: a case comparison trial

**DOI:** 10.1186/1471-2431-14-149

**Published:** 2014-06-16

**Authors:** Ron K Tam, Hubert Wong, Amy Plint, Nathalie Lepage, Guido Filler

**Affiliations:** 1 Departments of Pediatrics and Emergency Medicine, University of Ottawa, 401 Smyth Road, Ottawa, ON K1H 8L1, Canada; 2 Department of Pediatrics, Rouge Valley Health Center, 2867 Ellesmere Road, Toronto, ON M1E 4B9, Canada; 3 Department of Pathology and Laboratory Medicine, University of Ottawa, 401 Smyth Road, Ottawa, ON K1H 8L1, Canada; 4 Department of Pediatrics, Western University, 800 Commissioners Road East, London, ON N6A 5W9, Canada

**Keywords:** Gastroenteritis, Dehydration, Cystatin C, Microalbumin/creatinine ratio, Bicarbonate

## Abstract

**Background:**

The clinical dehydration scale (CDS) is a quick, easy-to-use tool with 4 clinical items and a score of 1–8 that serves to classify dehydration in children with gastroenteritis as no, some or moderate/severe dehydration. Studies validating the CDS (Friedman JN) with a comparison group remain elusive. We hypothesized that the CDS correlates with a wide spectrum of established markers of dehydration, making it an appropriate and easy-to-use clinical tool.

**Methods:**

This study was designed as a prospective double-cohort trial in a single tertiary care center. Children with diarrhea and vomiting, who clinically required intravenous fluids for rehydration, were compared with minor trauma patients who required intravenous needling for conscious sedation. We compared the CDS with clinical and urinary markers (urinary electrolytes, proteins, ratios and fractional excretions) for dehydration in both groups using receiver operating characteristic (ROC) curves to determine the area under the curve (AUC).

**Results:**

We enrolled 73 children (male = 36) in the dehydration group and 143 (male = 105) in the comparison group. Median age was 32 months (range 3–214) in the dehydration and 96 months (range 2.6-214 months, p < 0.0001) in the trauma group. Median CDS was 3 (range 0–8) within the dehydration group and 0 in the comparison group (p < 0.0001). The following parameters were statistically significant (p < 0.05) between the comparison group and the dehydrated group: difference in heart rate, diastolic blood pressure, urine sodium/potassium ratio, urine sodium, fractional sodium excretion, serum bicarbonate, and creatinine measurements. The best markers for dehydration were urine Na and serum bicarbonate (ROC AUC = 0.798 and 0.821, respectively). CDS was most closely correlated with serum bicarbonate (Pearson r = -0.3696, p = 0.002).

**Conclusion:**

Although serum bicarbonate is not the gold standard for dehydration, this study provides further evidence for the usefulness of the CDS as a dehydration marker in children.

**Trial registration:**

Registered at ClinicalTrials.gov (NCT00462527) on April 18, 2007.

## Background

Dehydration associated with gastroenteritis represents one of the leading causes of admission and morbidity in the pediatric emergency department (ED) [1]. It is also the most common cause of electrolyte abnormalities in children presenting at the ED [1,2]. In Canada, acute gastroenteritis accounts for 240,000 annual pediatric visits to the ED [3], while globally, diarrheal disease is responsible for approximately 10% of deaths in children under 5 years of age [4]. Considering its extensive global impact, it is not surprising that there are several serious complications associated with severe dehydration including hypo-volemic shock, pre-renal acute kidney injury, and acute tubular necrosis. Clinicians must determine whether patients only need to be rehydrated or whether they face more substantial morbidity, which can be challenging. Consequently, there has been considerable interest in developing a simple, non-invasive tool for measuring the severity of dehydration in children. Although previous studies have attempted to validate markers of dehydration by assessing the severity of dehydration using serial measurements of patient body weight [5,6], serial weights in sick and dehydrated children may be unreliable due to a number of factors that are not related to the severity of their illness. The clinical dehydration scale (CDS, Table 1) has been developed to meet this important objective [7,8]. The CDS combines scores of general appearance, eyes, mucous membranes, and tears. Use of the CDS has increased and it has been validated in 3 prospective studies, including one in the original ED [7], in a different Canadian pediatric ED [9], and in a multicenter trial at 3 Canadian EDs [10].

**Table 1:** Clinical dehydration scale for children with acute gastroenteritis used for the study

**Characteristic**	**Score of 0**	**Score of 1**	**Score of 2**
General appearance	Normal	Thirsty, restless, or lethargic, but irritable when touched	Drowsy, limp, cold, or sweaty; comatose or not
Eyes	Normal	Slightly sunken	Very sunken
Mucous membranes (tongue)	Moist	Sticky	Dry
Tears	Tears	Decreased tears	Absent tears

The CDS consists of four clinical characteristics (general appearance, eyes, mucous membranes, and tears), each of which are scored 0, 1, or 2 for a total score of 0 to 8, with 0 representing no dehydration; 1 to 4, some dehydration; and 5 to 8, moderate/severe dehydration. This score has been validated externally and is robust. We used exactly the same criteria as Benoit Bailey et al., Academic Emergency Medicine, 2010:17(6):583-88.

Following the development of the scale in 2004, Goldman et al., the originators of the scale, were first to attempt to validate the scale in a paper published in 2008 [7]. Their prospective observational study consisted of 205 children between 1 month and 5 years of age with suspected acute gastroenteritis. Since the original scale was developed using children 1–36 months of age, the aim of this study was to test this scale in a new cohort of children. Although the investigators found the dehydration categories of the scale to have a statistically significant correlation with length of stay (LOS) from time of arrival in triage and intravenous (i.v.) fluid rehydration, this study had numerous limitations: (i) it was only conducted in one center; (ii) it had a small number of children with moderate/severe dehydration; (iii) using LOS as an endpoint is questionable because LOS is multifactorial; (iv) staff may have changed their practices because of the study (Hawthorne effect), and, most importantly; (v) only a small number of the study population had blood tests performed, so the team could not validate their hypothesis that the dehydration categories positively correlated with abnormal serum pH values or bicarbonate levels (a primary outcome of the study). They indicated that future research is needed to provide information on this hypothesis.

A second study attempting to validate the CDS in a different emergency department was published in June of 2010 [9]. With 150 patients from 1 month to 5 years of age diagnosed with gastroenteritis, enteritis, or gastritis, the primary outcome of this study was LOS after being seen by the attending physician and the perceived need for IV fluid administration. Although serum bicarbonate and CO_2_ were measured, this was one of several secondary outcomes. Here, the correlation was statistically significant between the CDS and LOS from seeing the physician, perceived need for IV rehydration, and utilization of laboratory blood tests. Measured serum bicarbonate and CO_2_ were not found to significantly vary between the categories. Once again, this study had multiple limitations, the most important being that LOS is multifactorial, and although this was measured from the time the patient saw the physician, confounding factors may have still played a role.

Last, Gravel et al. [10] performed a multicenter validation of the CDS, published a few months later in October 2010. 264 children between the ages of 1 month and 5 years were recruited at 3 Canadian centers, presenting for acute vomiting and/or diarrhea. The primary outcome of this study was percent dehydration (difference in weight), while secondary outcomes included proportion of blood test measurements, IV use, hospitalization, and inter-rater agreement. This study found a statistically significant correlation between the CDS and percent dehydration (by weight), number of blood test measurements, IV rehydration use, hospitalization, and abnormal plasma bicarbonate. This study was limited in that it did not exclusively include patients with a gastroenteritis diagnosis, though a subgroup analysis was performed producing similar results, and the primary outcome could not be measured in 45 (17%) of patients. Finally, the use of percent dehydration is limited by certain confounders.

Although these studies have further validated this measure of dehydration, the primary outcome has differed in each study and all possess limitations (particularly LOS), none have employed the use of a comparison group (all 3 studies used a CDS score of 0 – “no dehydration” – for baseline measurements rather than a separate, non-dehydrated group), nor have they included a wide array of surrogate markers. The limitations of the preceding studies suggest the need for additional tests of validity for the CDS using other clinical markers.

We prospectively compared several established and novel markers of dehydration in two cohorts of children: a gastroenteritis group with dehydration and a comparison group without dehydration. Measuring the biomarkers in a comparison group provided baseline values and allowed us to validate the biomarkers in a healthy population prior to validating them in the dehydration cohort. The comparison group was comprised of patients with minor musculoskeletal injuries who were otherwise well and who required intravenous access for procedural treatment. We intended to validate the CDS by testing whether it correlates with certain factors, including bicarbonate, sodium, and others, and confirming its superiority to clinical impression.

## Methods

This study was designed as a case comparison trial and was registered at ClinicalTrials.gov (NCT00462527). It was conducted in a single center tertiary care pediatric emergency setting in Eastern Ontario. The study was supported through a grant to RT and GF from the Physicians’ Services Incorporated Foundation. Data used for this study was originally collected during a trial devised to examine the role of cystatin C as a biomarker of renal dysfunction in children with dehydration. Results were obtained from a secondary analysis of this data. Following approval by the Children’s Hospital of Eastern Ontario Research Ethics Board, written informed consent was obtained from patients (consenting minors) and caregivers. Patient enrollment took place between May 2007 and April 2008. All eligible pediatric patients (<18 years old) who consented were included in the study.

A dedicated research nurse screened patients for eligibility. All children presenting with vomiting, diarrhea, and dehydration who required laboratory testing as part of their medical care as decided by the most responsible physician (MRP, n = 17) were eligible for the experimental group. The comparison group comprised all children treated for musculoskeletal injury in the emergency department who required an intravenous line for conscious sedation or fracture reduction. Patients previously diagnosed with kidney disease, thyroid disease or chronic steroid use, who had undergone prior treatment for the same illness, or who chose not to participate in the study were excluded. Also excluded were patients with a head injury or abdominal (especially renal) trauma since this could affect their sodium handling or their tubular function.

The patient chart was used to obtain clinical, anthropometric, and demographic data, as described below. All serum tests were performed at intravenous needling. Urine tests were carried out on the earliest available urine from the patient and acquired either with a mid-stream voiding sample or a urine bag. The clinical definition for dehydration is the loss of body water, with or without salt, at a rate greater than the body can replace it; it is diagnosed through laboratory testing and clinical assessment. As there is no single standardized laboratory marker or laboratory score, we used a validated clinical scoring system. The attending MRP conducted scoring for the CDS [7,8]. The CDS consists of 4 clinical signs (general appearance, eyes, mucous membranes, and tears) individually scored between 0 and 2 for a total possible score out of 8. Each clinical sign of the CDS was chosen based on the results of a formal measurement methodology that assessed validity, reliability, discriminatory power, and responsiveness to clinical change, as published by Friedman et al [8]. All tests and measurements were obtained with the assistance of the dedicated research nurse. Inter- and intra-observer error was not assessed as there were no discrepancies between the rater assessments and independent assessments of the dedicated research nurse. The MRP was also asked to assess the degree of dehydration based on a scale of hydrated, mild, moderate and severe dehydration using his or her own clinical experience. These categories roughly reflected the level of dehydration according to body weight (5%, 10% or 15% respectively for younger than 2 years old or 3%, 6% or 9% for older than 2 years old). Following this procedure, the patient continued to receive treatment independent of the study and care was directed by the MRP.

Clinical data recorded included: gender, age, length of illness, duration of oligo-/anuria, date of admission to hospital, final discharge diagnosis, and need for dialysis. Anthropometric measurements were obtained as a part of routine clinical practices and included height and weight (measured using a high-precision industrial scale [Scale-Tronix scales 6002 for wheelchair patients, 4802 for infants and 5002 otherwise, Scale-Tronix, Wheaton, Illinois, USA]). Blood pressure measurements were taken sporadically using a standardized protocol employing automated oscillometric blood pressure machines (patient seated, calm, second of two measurements performed 5 minutes apart with either Walch Allyn Spot Vital Signs LXi [Walch Allyn, Skaneateles Falls, New York, USA], or Dinamap Pro 100, Pro 300 and Dinamap XL Vital Signs Monitor, [Criticon, Tampa, Florida, USA]). Additional laboratory data included serum and urine electrolytes, urea, serum bicarbonate and creatinine (Ortho Clinical Diagnostics), osmolality (Advanced Instruments), urine alpha-1 microglobulin (a low molecular weight protein) and urine microalbumin (Beckman-Coulter), and serum cystatin C (Dade-Behring).

### Calculations and statistical analysis

Glomerular filtration rate was calculated using the serum creatinine formula published by Schwartz [11] and the cystatin C formula published by Filler [12]. Fractional excretion of sodium (FeNa) and urea (FeUrea) were calculated using the following standard formula:

$$\frac{\text{sodium}\;\mathrm{or}\;\text{ure}a_{\text{urine}}\;\left(\frac{\text{mmol}}{L}\right)*\text{creatinin}e_{\text{plasma}}\;\left(\frac{\mu \text{mol}}{L}\right)}{\text{sodium}\;\mathrm{or}\;\text{ure}a_{\text{plasma}}\;\left(\frac{\text{mmol}}{L}\right)*\text{creatinin}e_{\text{urine}}\;\left(\frac{\mu \text{mol}}{L}\right)}$$

The ratio urine Na/K was calculated using the following standard formula:

$$\frac{\text{sodiu}m_{\text{urine}}\left(\frac{\text{mmol}}{L}\right)}{\text{potassiu}m_{\text{urine}}\left(\frac{\text{mmol}}{L}\right)}$$

Percent dehydration was calculated using:

$$\frac{\text{final}\;\text{weight}-\text{initial}\;\text{weight}}{\text{final}\;\text{weight}}\times 100$$

Weight and blood pressure z-scores were calculated using the methodology provided by the Centers for Disease Control (CDC) website, with age and gender matched controls taken from the National Centre for Health Statistics (USA) using the published Box Cox transformations [13-16]. The most recent National Health and Nutrition Examination Survey (NHANES) III database (1999–2002) was used for all patients [NCHS (National Center for Health Statistics) – 2000 CDC Growth Charts: United States (Accessed July 29, 2006, at http://www.cdc.gov/growthcharts/)]. The GraphPad Prism (Version 4.03, GraphPad Prism Software for Science, San Diego, CA, USA) and MedCalc (Version 11.0.1.0, MedCalc Software bvba, Broekstraat 52, 9030 Mariakerke, Belgium) statistical packages were used for statistical analysis. Contiguous data were analyzed for normal distribution using the Shapiro-Wilk normality test. Mean and standard deviation were reported for normally distributed data; otherwise, median and quartiles were given. Comparisons were first made between cohorts to identify statistically significant biochemical and physical markers of dehydration. All statistically significant markers were then compared with receiver operating characteristic (ROC) curves to determine the marker with the highest sensitivity and specificity for the binary outcome of the presence or absence of dehydration as per the initial screening. Data collected from the comparison group served as the gold standard in relation to the dehydration group. Any area under the curve (AUC) greater than 0.8 was considered significant. Next, markers of dehydration and CDS were compared using linear and non-linear correlation curves. A two-tailed p value of 0.05 was considered significant where applicable. No adjustment was made for missing data.

## Results

**Figure 1:**
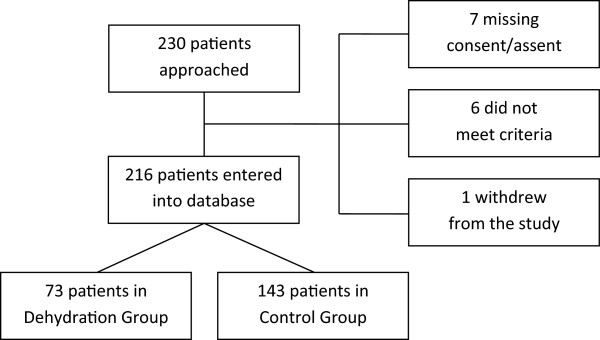
Flow diagram of patients’ enrollment.

230 patients were approached between May 2007 and April 2008 to participate in the study. Fourteen patients could not be enrolled for various reasons (seven did not meet the criteria, six were missing assent/consent, and one withdrew early into the study), leaving 216 patients. Seventy-three children were enrolled in the dehydration group. Thirty-six patients were male (49.3%) with a median age of 32.5 months (range 3.3 to 214 months). Additionally, 143 patients (105 male children, 73%) were enrolled in the comparison group with a median age of 96 months (range 2.6 to 214 months) (Figure 1).

Complete data were available for hydration assessment, clinical hydration score, pre-hydration weight, and serum sodium, potassium, and chloride. Nearly complete data (<5% missing) were available for pre-hydration blood pressure, blood urea, and serum bicarbonate, creatinine, osmolality, albumin, and cystatin C. Post-hydration blood pressure and post-hydration weight was available for 90% of patients, while urine tests were available for 88% of patients. In total, 90.22% of data were complete.

The most common cause of dehydration was viral gastroenteritis (n = 59, 80.8%). Other causes included pneumonia (n = 1), appendicitis (n = 2), cellulitis (n = 1), hemolytic uremic syndrome (not oliguric, n = 1), and unspecified (n = 9). Importantly, other than viral gastroenteritis, the patients were not diagnosed when they were screened and clinically, they all appeared as dehydrated patients. Patients in the comparison group requiring conscious sedation were most commonly diagnosed with fractures (n = 129).

Patient evaluations yielded the following dehydration scores: none to mild (Friedman CDS 0–1): 13; mild to moderate (Friedman CDS 2–3): 27; moderate (Friedman CDS 5–6): 27; and severe (Friedman CDS 7–8): 6. Following assessment, it was determined that all patients in the comparison group were hydrated. The median CDS score in the dehydration group was 3 (range 0 to 8). Every patient in the comparison group scored 0 on the same scale. There was a close correlation between the dehydration score of the MRP (median 3, range 1–4) and the CDS (r = 0.60, p < 0.0001). Given that the median clinical impression MRP score of 3 was at the higher end of the scales whereas the median CDS score of 3 was at the relatively milder end of the dehydration spectrum, clinicians’ impressions appear to overestimate the degree of dehydration.

As expected, patients in the dehydrated group were more tachycardic and had an elevated diastolic z-score when compared with the comparison group, although this did not reach statistical significance. Following treatment, systolic, diastolic and heart rate z-scores declined in the dehydrated group in response to fluid treatment (two-tailed paired t-test p = 0.04, p < 0.0001, and p < 0.0001, respectively). Results are summarized in Table 2. Of note, there were missing values for the post-rehydration weights. Only 43 patients in the dehydration group and 103 patients in the comparison group had both a pre- and post-hydration weight. Weight z-score was normally distributed. The mean weight z-score prior to rehydration (0.271 ± 1/25) and the post-hydration z-score (0.154 ± 1/511, n = 43, p = 0.4355, paired t-test) were not significantly different in the dehydration group, while the pre-intervention weight (0.445 ± 0.951) and the post-intervention weight z-score (0.435 ± 1.098, n = 103, p = 0.8567, paired t-test) were not significantly different in the comparison group. There was also no significant difference in the weight z-score between both groups (p = 0.360 and 0.212 for the pre- and post-intervention weight z-scores, respectively).

**Table 2:** Demographic and physical examination data of dehydration and comparison group, pre- and post-treatment

		**Dehydration n = 73**	**Comparison n = 143**	**P value**
**Number of patients**		73	143	
**Number of males (%)**		36 (49.3%)	105 (73%)	0.0008*
**Age (months)**		32.5 (3.3-214)	96 (2.6-214)	<0.0001†
**Pre-treatment**				
	Weight z-score	0.24 ± 1.27	0.50 ± 1.07	0.18
	Systolic z-score	0.98 ± 1.0	1.19 ± 1.2	0.20
	Diastolic z-score	1.33 ± 1.1	0.68 ± 1.0	<0.0001
	HR z-score	1.0 ± 1.1	-0.06 ± 1.3	<0.0001
**Post-treatment**				
	Weight z-score	0.15 ± 1.51	0.41 ± 1.11	0.26
	Systolic z-score	0.72 ± 1.0	1.3 ± 1.3	0.0025
	Diastolic z-score	0.82 ± 1.1	0.65 ± 1.0	0.293
	HR z-score	-0.04 ± 1.2	-0.3 ± 1.3	0.16
**Clinical dehydration Score (0–8)**		3 (0–8)	0	<0.0001‡
**Percent dehydration (%)**		1.2 (-8.2-8.6)	0.6 (-2.5-7.3)	0.61

HR = heart rate, bpm = beats per minute, Percent dehydrated = (post-weight- pre-weight)/ post weight *100. All data is expressed as mean ± standard deviation or median (range), depending on the results of the normality test (Shapiro-Wilks).

*= Fisher’s exact test, † = Mann Whitney test, ‡= Wilcoxon signed rank test, all others unpaired.

**Figure 2:**
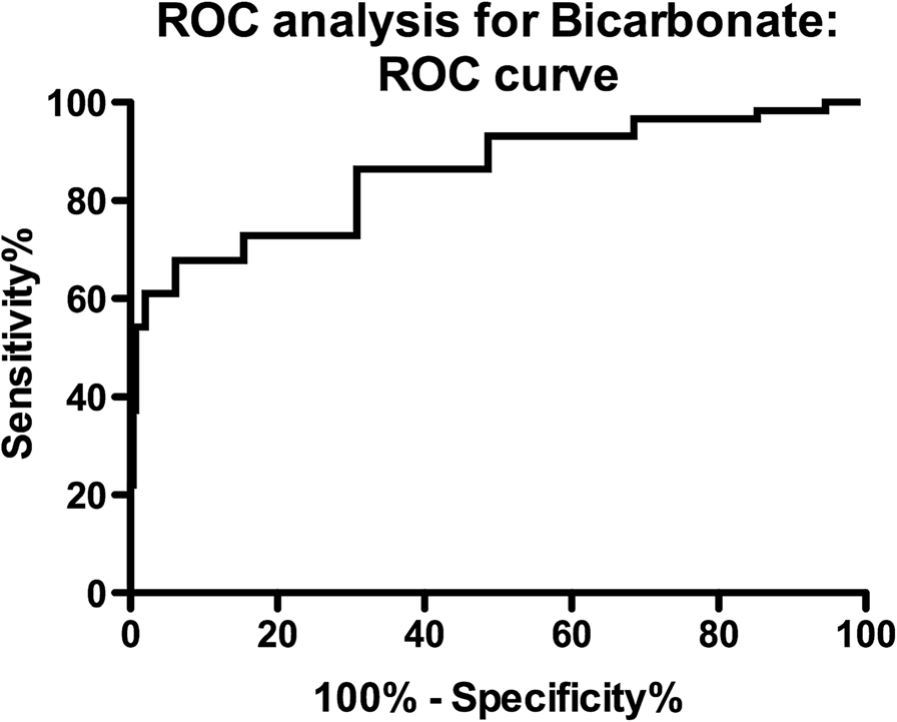
**Serum bicarbonate correlates well with severity of clinical dehydration score (p=0.0027, r - 0.355, R-squared 0.1262).** A serum bicarbonate of 21 mmol/L has a sensitivity of 90% and a specificity of 62% for dehydration in children and is most closely associated with a score of 2 or greater (dotted line). Confidence intervals are represented with dashed line.

**Figure 3:**
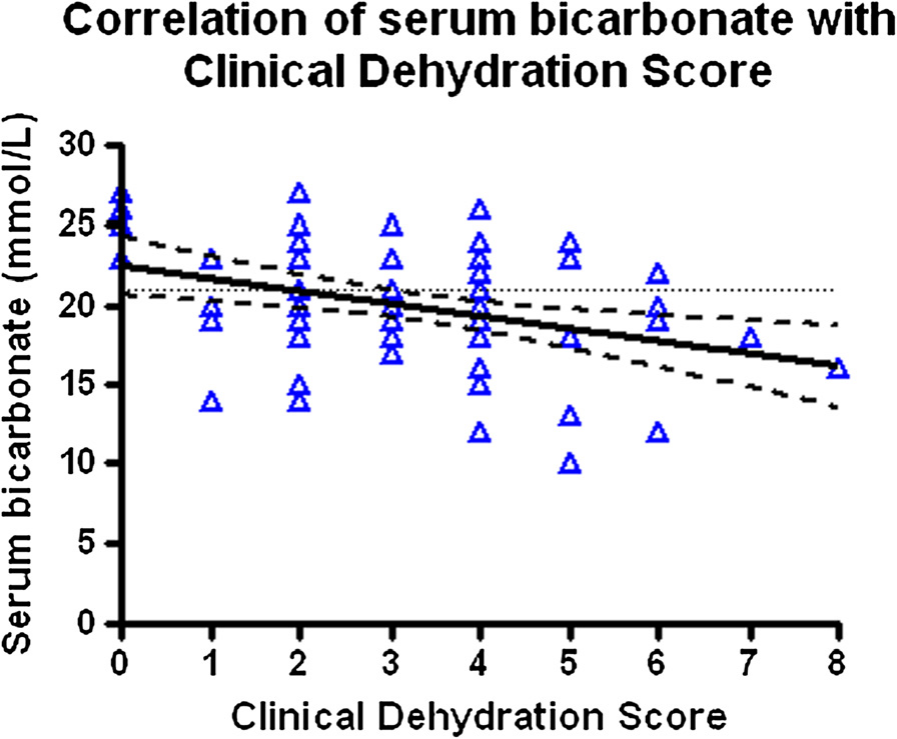
**Received operating characteristic plot for serum bicarbonate to determine the predictability of serum bicarbonate and CDS for the presence or absence of dehydration.** AUC was 0.821 (95% confidence interval 0.79 to 0.92).

As hypothesized, urine Na/K ratio (p < 0.0001), urine Na (p < 0.0001), FeNa (p < 0.0001), blood urea (p = 0.01), and serum bicarbonate (p < 0.0001) and creatinine (p = 0) were all significantly different between both groups (Table 3). Serum cystatin C (p = 0.58),% dehydration by body weight (p = 0.61), FeUrea (p = 0.66), urine osmolality (p = 0.2), and serum osmolality (p = 0.11) did not reach statistical significance. Both the urinary microalbumin (p < 0.0001) and urinary alpha-1 microglobulin (p < 0.001) also reached a high statistical significance level.We performed ROC analysis to compare sensitivity and 1-specificity between both groups. The binary outcome of interest for the ROC analysis was the presence of absence of dehydration per initial screening. Serum bicarbonate recorded the highest AUC (0.821 95% confidence interval 0.79 to 0.92, Figure 2). Urine Na of less than 90 mmol/L had a sensitivity of 75% and specificity of 74% (p = 0.0001) and serum bicarbonate of less than 21 had a sensitivity of 90% and a specificity of 62% for dehydration in children with diarrhea and/or vomiting (p = 0.001).To validate the CDS, we performed correlation analysis. There was a significant negative correlation between serum bicarbonate and the severity of CDS and hydration assessment (Figure 3). A CDS score of 2 or greater was roughly associated with a serum bicarbonate of 21 mmol/L or less. None of the other biochemical or physical markers of hydration correlated with the CDS.

**Table 3:** Comparison of various markers of dehydration in two cohorts

**Markers**	**Dehydrated n = 73**	**Comparison n = 143**	**AUC (SE)**	**P value**
**Dehydration score**	3 (0–8)	0 (0–0)		<0.0001
**Pre-hydration systolic blood pressure z-score**	0.57 (-0.18-1.54)	0.97 (0.02-1.90)		n.s.
**Pre-hydration diastolic blood pressure z-score**	0.67 (-0.09-1.21)	0.24 (-0.42, 1.10)		n.s.
**Pre-hydration heart rate**	134 (112, 158)	97 (84, 130)		<0.0001
**Urine Na/K ratio **[17]	0.69 (0–4.4)	2.3(0–56)	0.789(0.03)	<0.0001
**Serum Osmolality [mOsm] **[18]	286 (231–684)	289 (199–391)		0.11
**Urine Na [mmol/L] **[19]	66.0 ± 55.6	144 ± 74	0.798(0.03)	<0.0001
**FeNa **[20]	0.19 (0–0.89)	0.52 (0–10.4)	0.753(0.03)	<0.0001
**FeUrea **[21]	0.398 (0.678-0.96)	0.377 (0.055-4.504)		n.s.
**Serum bicarbonate [mmol/L]**	20 (10–27)	24 (18–30)	0.821(0.03)	<0.0001
**Blood urea [g/L]**	5.7 (1.2-41)	5 (2.6-9.2)	0.613(0.05)	0.01
**Serum creatinine [μmol/L]**	38 (16–408)	45 (3.8-97)	0.666(0.04)	0
**Schwartz eGFR [mL/min/1.73 m**^**2**^**]**	131.6 ± 32.3	136.9 ± 23.2		0.18
**Cystatin C [mg/L]**	0.67 (0.43-2.89)	0.66 (0.44-1.08)		n.s.
**Cystatin C eGFR [mL/min/1.73 m**^**2**^**]**	144.0 ± 36.9	147.6 ± 25.1		n.s.
**Urine osmolality [mOsm]**	805 ± 306	876 ± 402		0.2
**Urinary microalbumin/creatinine ratio [mg/mmol] **[22]	4.4 (0.4-61.1)	2.3 (0.3-9.4)	0.69 (0.04)	<0.0001
**Urinary α1-microglobulin/creatinine ratio [mg/mmol] **[22]	1.75 (0.30-14.70)	0.70 (0.20-11.30)	0.809(0.04)	P < 0.001

Student’s t-test or Mann Whitney test of established and potential markers of dehydration and acute kidney injury with key references listed. Data are expressed as mean and one standard deviation or median and range, as appropriate based on the distribution. “n.s.” means not significant. Results are expressed as median (range) or mean ± standard deviation where applicable. Urine Na/K ratio, urine Na, FeNa, serum bicarbonate, serum creatinine and blood urea were all significantly different. FeNa = [urine sodium (mmol/L) × plasma creatinine (μmol/L)] / (plasma sodium (mmol/L) ÷ urine creatinine(μmol/L)] × 100. AUC = Receiver operating characteristic curves area under the curves. SE = standard error.

Ten patients in the dehydration group were admitted to receive ongoing treatment. Their CDS ranged from 0 to 6. Although statistical analysis was not performed on this small cohort, there was no apparent relationship between the severity of CDS and whether patients were admitted to hospital or discharged from the emergency department. One patient in the dehydration group suffered from hemolytic uremic syndrome and required acute dialysis for 3 days. This patient scored a 5 on the CDS.

## Discussion

Since there is considerable uncertainty in this area, assessing a dehydrated patient and accurately determining the severity of his or her dehydration remains a challenge in the pediatric emergency department. The current study represents the first attempt to independently assess the diagnostic performance of established biochemical surrogate markers of dehydration such as fractional excretion of sodium or urine Na/K ratio against the Friedman CDS. The CDS was developed using percent dehydration based on measured weights and was validated against three criteria: LOS in hospital, the need for intravenous rehydration and serum bicarbonate [7,8]. As discussed in their report, both LOS and the need for intravenous rehydration are subjective parameters influenced by a number of factors including the severity of the patient’s illness. For example, LOS may be affected by bed access, local practices, family preference, and the demands of the nursing resources in the emergency department, while physician seniority and training and the need to manage emergency space quickly and efficiently often influence decisions related to intravenous treatment. Furthermore, concrete laboratory parameters such as serum bicarbonate have been linked to the severity of dehydration [2]. Vega et al. have demonstrated that in addition to serum urea increasing, serum bicarbonate declines with increasing percentage of lost body weight [6]. The current study also confirmed more urea in our patients, although the degree of change was modest and not clinically significant. Surprisingly, the present study did not demonstrate a significant association between the fractional urea excretion [23] that is rarely studied in children in this setting.

This study points to an association between serum bicarbonate and a patient’s score on the dehydration scale, thereby indirectly validating the CDS. This result is supported by Gravel et al. [10], although findings comparing the CDS and serum bicarbonate have been inconsistent [7,9]. Serum bicarbonate was shown to have the highest sensitivity and specificity to predict dehydration. In contrast, we found no relationship between hospital admission rate and CDS score, most likely because hospital admissions reflect a number of factors beyond the severity of illness on presentation. For example, the ability to provide adequate follow-up care, the patient’s proximity to the hospital, and response to treatment also influence hospitalization. Other measurable outcomes such as acute kidney injury, assessed using RIFLE criteria [24], occurred only once and were too infrequent to analyze.

The present study examines two components not previously addressed in current literature. First, our selection criteria biased our dehydration group to children with more severe disease. By limiting our recruitment strategy to only include patients who required intravenous needling, we anticipated greater differences between the dehydration and comparison groups and an increase in the likelihood of complications associated with dehydration. This also strengthened the utility of the results of the laboratory tests, since they are more likely to be helpful in determining hydration when results are markedly abnormal [25]. Second, we included a hydrated cohort to strengthen our analysis. Although age and gender differed between the two cohorts, it is important to note that even though the CDS measure was originally developed for use in children <3 years of age, that same center conducted a validation study in children 1 month to 5 years old and subsequent validation studies have included children up to 5 years of age [7,9,10], suggesting the usefulness of the scale in children up to 5 years of age. We also accounted for age bias by using age- and gender-independent z-scores.

In total, six biochemical and two clinical parameters distinguished dehydrated patients from the comparison group. As expected, these included: diastolic blood pressure z-score, heart rate z-score, urine Na/K ratio, urine Na, FeNA, blood urea and serum bicarbonate and creatinine. It should be noted that the urea differences, albeit significant, were not clinically relevant. Unforeseen, however, were the categories that did not identify children with dehydration. These included percent dehydration and urine osmolality. Numerous studies have used percent dehydration as a gold standard to quantify the degree of dehydration in a child [5,26], Unfortunately, despite the assistance of trained and dedicated research nurses to perform and ensure adequate and consistent weight measurement prior to and following treatment, the difference in percent dehydration did not reach statistical significance. This further supports the subjectivity of this parameter despite employing specific training. Furthermore, other factors that contribute to weight gain or loss during an acute illness episode may have influenced these findings, including the amount of rehydration, decreased intake, ongoing oral/rectal and urinary losses, increased insensible losses and an increased catabolic rate. Additionally, intravascular volume may be the most important factor in complications associated with dehydration such as hypo-volemic shock or acute kidney injury. Severe dehydration requires prompt restoration of intravascular volume through intravenous administration of fluids followed by oral rehydration therapy [27]. Body water movement from compartment to compartment during any time period can be attributed to forces active within and upon each space. These forces lead to water transfer between intravascular and extravascular compartments and shifts between extracellular and intracellular spaces [28], and may be independent of body weight.

In previous studies, post-hydration weight was measured up to one week following the illness episode. However, this approach has limitations since serial weight measurements are both difficult to obtain and may not yield  healthy’ weights in the time they are measured. For example, Gorelick et al. reported on the reliability of clinical signs in 186 children but only 77 had stable reliable  healthy’ weights measured following enrollment [5]. Findings based on data from these 77 patients were then extrapolated to the entire group of 186 children [5]. Friedman et al. and Gravel et al. also based the development of the CDS on the serial measurements of  healthy weights’. However, pre- and post-hydration weights were only available from 102 of 141 (74%) children enrolled in the study by Friedman et al. [8], and 83% were available in the study by Gravel et al. [10]. Finally, Teach et al. continued to observe a further decline in the  healthy’ weights in 12.5% of follow-up patients who were re-examined between 24 hours and 7 days post-treatment [26]. Our own data shows a further decline in weight at time of discharge in 23% of patients. Clearly, the reliability of using serial weights to validate the severity of dehydration in children has limitations. It is for this reason that we believe employing the use of a hydrated cohort as a comparison group is a more reliable method of assessing markers of dehydration. However, it is debatable whether or not a CDS of 2 or more is better than the subjective rating of dehydration.

The current study has several limitations, including the difference in age between the dehydrated group and the comparison group. This may have influenced the difference in serum creatinine concentrations seen between the two groups, although we corrected for this by using the Schwartz formula to estimate eGFR per body surface area. Heart rate may also differ by age. Additionally, we recruited a relatively small number of patients with severe gastroenteritis. Study inclusion criteria and early parental intervention for sick children may have played a role in recruiting these patients. Also, we did not formally assess the inter- and intra-observer error for the CDS score. The use of early oral antiemetic medication (eg. odansetron) has reduced the amount of intravenous rehydration and thus decreased the number of patients eligible for recruitment into the dehydration group [29]. Furthermore, we only had post-hydration weights for 60% of patients. We also included some patients with a CDS of zero which should be considered “not dehydrated”. The high urinary osmolality in the comparison group might suggest that these patients were in fact not well hydrated, even though their clinical CDS was zero. Importantly, the CDS scoring system was developed for children <5 years of age and our reference group was older. The CDS score has not been validated in older children.

## Conclusion

Since assessing a dehydrated patient and accurately determining the severity of his or her dehydration remains a challenge in the pediatric emergency department, there has been considerable interest in creating a non-invasive tool such as a validated scale to measure this parameter. Thus, this case comparison trial was designed to validate the Friedman CDS, a tool which can be used to meet this objective. The study found that a CDS score of 2 or greater was associated with serum bicarbonate of 21 mmol/L or less, which provides further evidence for the usefulness of the CDS as a dehydration marker in children.

## Abbreviations

AUC: Area under the curve; CDC: Centers for Disease Control; CDS: Clinical dehydration score; ED: Emergency department; MRP: Most responsible physician; NCHS: National Center for Health Statistics; NHANES: National Health and Nutrition Examination Survey; ROC: Receiver operating characteristic.

## Competing interests

The authors declare that they have no competing interests.

## Authors’ contributions

RT, HW and GF (as principal investigator) conceived the idea for the study, wrote the grant proposal to Physician Services Incorporated, and were responsible for the overall study. RT was responsible for patient recruitment in the emergency room, organized the study among colleagues in the emergency room, collected the data and wrote the draft manuscript. HW performed the calculations for the study, and helped with each version of the manuscript. AP also recruited patients, provided valuable feedback at all stages of the development of the manuscript, and provided scientific rigor throughout the process of the study. NL organized all laboratory measurements, reviewed all stages of the manuscript, and was responsible for the smooth operation of the laboratory part of the study. GF mentored the junior faculty, supervised the study, applied for research ethics board approval, performed and verified all analyses, and was responsible for the overall study. All authors read and approved the final manuscript.

## Pre-publication history

The pre-publication history for this paper can be accessed here:

http://www.biomedcentral.com/1471-2431/14/149/prepub
